# Health Behaviours and the Quality of Life of Students of Medical Fields during the COVID-19 Pandemic

**DOI:** 10.3390/nu16111747

**Published:** 2024-06-02

**Authors:** Ewa Kupcewicz, Daria Schneider-Matyka, Kamila Rachubińska, Paweł Jastrzębski, Aleksandra Bentkowska, Elżbieta Grochans

**Affiliations:** 1 Department of Nursing, Collegium Medicum, University of Warmia and Mazury in Olsztyn, 14 C Zolnierska Street, 10-719 Olsztyn, Poland; ekupcewicz@wp.pl; 2 Department of Nursing, Pomeranian Medical University in Szczecin, 48 Żołnierska Street, 71-210 Szczecin, Poland; kamila.rachubinska@pum.edu.pl (K.R.); grochans@pum.edu.pl (E.G.); 3 Department of Emergency Medicine, Collegium Medicum, University of Warmia and Mazury in Olsztyn, 14 C Żołnierska Street, 10-719 Olsztyn, Poland; pawel.jastrzebski@uwm.edu.pl; 4 Hospital Emergency Department, Provincial Specialist Hospital in Olsztyn, 18 Żołnierska Street, 10-719 Olsztyn, Poland; a.bentkowska.kruszwicka@gmail.com

**Keywords:** health behaviours, quality of life, COVID-19, students

## Abstract

(1) Background: Social distancing and closing down public spaces associated with learning, leisure and physical activity limited the spread of COVID-19. These measures had an impact not only on the economy and education but also on health behaviours and the quality of life of individuals affected by the restrictions. The aim of this study was to identify the role of health behaviours in the perception of the quality of life of students during the COVID-19 pandemic. (2) Methods: This study was conducted among 796 students of the University of Warmia and Mazury in Olsztyn in the first quarter of 2022. Subgroup 1 consisted of students at the Public Health School (*n* = 428; 53.8%) and subgroup 2 consisted of students belonging to the Faculty of Veterinary Medicine (*n* = 368; 46.2%). The diagnostic survey method was applied, and an original survey questionnaire, the Health Behaviour Inventory and the Quality of Life Questionnaire (WHOQoL-Bref version) were used. (3) Results: The largest contribution to the prediction of quality of life of students in subgroup 1 was made by a positive mental attitude related to avoiding too strong emotions, which explained 19% of the result variability in the somatic domain (ßeta = 0.24; R^2^ = 0.21), 20% of the result variability in the psychological domain (ßeta = 0.36; R^2^ = 0.20), 16% of the result variability in the social domain (ßeta = 0.52; R^2^ = 0.17) and 17% of the result variability in the environmental domain (ßeta = 0.19; R^2^ = 0.19). Moreover, in subgroup 2, a predominantly positive mental attitude significantly predicted quality of life in the somatic domain, explaining 23% of the result variability (ßeta = 0.24; R^2^ = 0.26), while it explained 25% of the result variability in the psychological domain (ßeta = 0.47; R^2^ = 0.25), 16% of the result variability in the social domain (ßeta = 0.46; R^2^ = 0.17) and 21% of the result variability in the environmental domain (ßeta = 0.38; R^2^ = 0.23). (4) Conclusions: Positive correlations between health behaviours and the quality of life among the study participants were determined. Health-promoting behaviours had a beneficial impact on the respondents’ quality of life during the COVID-19 pandemic. The category of health behaviours described as a positive mental attitude was an important predictor for the participants’ quality of life.

## 1. Introduction

Health behaviours are a combination of human knowledge, actions and attitudes adopted by an individual for reasons related to their psychophysical condition. They are also defined as all health-related activities—both pro-health and anti-health ones. Pro-health behaviours include habits, customs, actions, attitudes and values that are regarded as pro-health by members of society. These include regular exercise, healthy eating, sufficient amount of sleep, avoiding addictive substances and being able to cope with stress. Therefore, one can regard health behaviours as a system of beliefs, expectations, thoughts and motives related to health [1,2]. Health behaviours develop mainly during the second decade of one’s life and undergo transformation throughout it. They are passed on in the process of socialisation, and they emerge in the course of social interactions during childhood and youth under the influence of information passed on by one’s parents, guardians, school, media and healthcare professionals. The second decade of one’s life, which coincides with the beginning of university education, is the time for breakthroughs in health behaviours. The significant role of an individual’s personal and social resources, whose type and level can affect one’s health behaviours, is stressed increasingly often [3,4,5].

The pandemic related to COVID-19, which is a disease of high infectivity and clinical intensity, brought about a range of consequences, including the need for social distancing, which was the main measure aimed at preventing the virus’ spread [6]. Governments all over the world implemented a variety of preventive measures, which resulted in considerable changes in everyday life. The number of people who could remain inside shops was limited, many restaurants suspended their activities and access to recreation and sports facilities was closed as one of the main preventive measures. Due to the need for social distancing, academic activities were suspended at universities all over the world. Observing these measures by students meant numerous changes, such as distanced learning, being confined to their home, limited mobility, closed access to leisure facilities and physical exercise opportunities and hindered access to food. Restrictions imposed in connection with the COVID-19 pandemic had an impact on food consumption, sedentary lifestyles and physical exercise. Long-term social isolation could have a considerable impact on health behaviours and result in physical, mental, social and economic issues among university students, which could also have an impact on the quality of their lives [7]. A study by Luciano et al. observed reduced physical activity and increased sleep and inactivity time among medical students during the COVID-19 pandemic compared to the pre-pandemic period [8]. Similar findings were obtained in studies by other authors conducted in the general population. It was shown that the majority of respondents declared less activity during the isolation period than before the pandemic [9,10,11,12,13]. A study by Celorio-Sardà et al. showed a trend towards healthier eating habits [14]. In contrast, other studies indicated an increase in the weight of subjects during the period of social isolation [15,16,17,18]. In addition, some studies indicated a reduction in alcohol consumption among adolescents [19,20]. However, these reports are not consistent. Zysset et al. showed that a significant number of students developed more risky health behaviours related to alcohol consumption during the COVID-19 pandemic [21].

Research by Awad et al. indicated that the pandemic also negatively affected medical students’ quality of life, especially their mental health [22]. These findings have also been supported by studies conducted by other researchers under similar conditions [23,24,25,26,27]. According to the WHO, quality of life can be defined as an individual perceiving their life situation in the context of culture and their systems of values and in reference to their goals, expectations, standards and concerns. This is a broad term that covers links to the environment, physical and mental aspects, level of independence, social relations and personal beliefs [28,29,30].

According to study findings, social distancing and closing down public spaces associated with learning, leisure and physical activity limited the spread of COVID-19 [31,32]. These measures had an impact on the economy and education, but also on physical and mental health and on the quality of life of individuals affected by the restrictions [33,34,35]. There is a need to identify changes in students’ health behaviours linked to these restrictions in order to understand better the psychosocial consequences of the pandemic. Changes in health behaviours associated with lifestyle are largely unavoidable. These will probably include changes in sleep patterns, alcohol consumption, physical exercise and eating habits, which can determine quality of life [36].

The aim of this study was to identify the role of health behaviours in the perception of the quality of life of students at the University of Warmia and Mazury in Olsztyn during the COVID-19 pandemic.

The following research questions were formulated:

1. Are health behaviours and the quality of life among students differentiated during the COVID-19 pandemic, and if so, what is the extent of this differentiation?
2. Are there any correlations between health behaviours and the quality of students’ lives during the COVID-19 pandemic, and if there are, what is their extent, and which categories of health behaviours can play the role of predictors for the quality of life?

## 2. Materials and Methods

### 2.1. Settings and Design

Students at the University of Warmia and Mazury in Olsztyn were invited to take part in a survey carried out between January and March 2022. There were 796 students enrolled for the study, including 428 (53.8; subgroup 1) belonging to the Public Health School—medical majors, i.e., nursing, midwifery, emergency medicine and dietetics—and 368 (46.2%; subgroup 2) belonging to the Faculty of Veterinary Medicine, with the major of veterinary medicine. The enrolment criteria were that the students were under 30 years old and provided informed consent for participation in the study. Anyone who did not provide consent was excluded from the study. Researchers obtained the dean’s consent and conducted the survey in direct contact with the students while adhering to the sanitary regime. Participation in the study was voluntary and anonymous. The survey was conducted in groups of about a dozen students. The students were briefed on the study objective and given the chance to ask questions and receive answers. Participants were also informed that they had the option to withdraw from the study at any time. It took approximately 15 min to complete the questionnaire. Altogether, 850 questionnaire sets were distributed among the students, and 798 (93.9%) were included in the final analysis. The present study is part of a larger research project, which was approved (No. 3/2021) by the Senate Scientific Research Ethics Committee at Olsztyn University in Olsztyn. This study was conducted in accordance with the rules of the Helsinki Declaration.

### 2.2. Research Instruments

A diagnostic survey method was utilised, and the following research tools were employed to gather the data:

- An original questionnaire contained questions concerning demographic data, i.e., age, year of study, sex, place of residence during the pandemic, the extent of restriction of social contacts, number of hours of working from home, frequency of meals during the day, the extent to which physical exercise was limited and their preferred form of physical exercise;
- The Health Behaviour Inventory (IZZ) adapted for the Polish language by Z. Juczyński [37];
- The Quality of Life Questionnaire (version WHOQoL-Bref) adapted for the Polish language by L. Wołowicka and K. Jaracz [38].

#### 2.2.1. Health Behaviour Inventory

The IZZ questionnaire by Z Juczyński was used to assess the general intensity of pro-health behaviour and four categories of health behaviours:

- Proper eating habits (type of food eaten, e.g., wholemeal bread, fruit and vegetables);
- Prophylactic behaviour (following health-related recommendations, acquiring information on health and disease);
- Health practices (they include daily habits related to sleep, recreation and physical exercise);
- Positive mental attitude (avoiding too strong emotions, stress and tensions).

The questionnaire contained 24 statements describing various health-related behaviours. The respondents indicated the frequency of these health activities by rating them on a five-point Likert scale, from “hardly ever” (1 point) to “nearly always” (5 points). The points were then added together. The scores lay within the interval between 24 and 120 points. The higher the score, the higher the intensity of declared health behaviours. After being converted to standardised units, the overall index was interpreted according to the properties of the sten scale. Scores between 1 and 4 sten were regarded as low, scores 5 and 6 were regarded as average, whereas those from 7 to 10 sten were regarded as high. Moreover, the intensity of four categories of health behaviours was calculated. The index was defined as the mean score in each category. The questionnaire had good psychometric properties. The IZZ’s internal consistency based on Cronbach alpha was 0.85 for the whole scale, while it lay within an interval from 0.60 to 0.65 for its four subscales. The test–retest examination produced a correlation coefficient of 0.88 [37].

#### 2.2.2. WHOQoL-Bref Questionnaire

The WHOQoL-Bref questionnaire is an abbreviated version of WHOQoL-100 developed by a group of quality-of-life researchers at the WHO. It contains 26 questions and allows researchers to obtain a quality of life profile in terms of functioning in the following domains: somatic—seven questions (activities of daily living, dependence on medicinal substances and medical aids, energy and fatigue, mobility, pain and discomfort, sleep and rest, work capacity) [38,39,40]; mental—six questions (body image and appearance, negative feelings, positive feelings, self-esteem, religion, spirituality, personal beliefs, thinking, learning, memory, concentration) [35,36,37]; social—three questions (personal relationships, social support, sexual activity) [38,39,40]; and environmental—eight questions (financial resources, freedom/physical safety and security, health and social care: accessibility and quality, home environment, opportunities for acquiring new information and skills, participation in and opportunities for recreation and leisure, and physical environment, including pollution, noise, traffic, climate and transport) [38,39,40]. Two questions ask about satisfaction with the overall quality of life and satisfaction with the overall quality of health.

A participant provided answers based on the 5-point Likert scale ranging from 1 to 5, where 1 = very dissatisfied and 5 = very satisfied. A maximum of 20 points could be scored for each domain. The results for individual domains had a positive direction (the larger the number of points, the higher the quality of life). The questionnaire has good psychometric properties, and the reliability of the Polish version of the WHOQoL-Bref questionnaire is close to that of the original. The Cronbach alpha could be regarded as very high, both in the assessment of individual domains (between 0.69 and 0.81) and of the whole questionnaire (0.90) [38].

### 2.3. Statistical Analysis

The statistical analysis was performed using STATISTICA v.13.3 (TIBCO, Palo Alto, CA, USA). The variables were described using descriptive statistics methods, including arithmetic mean (M) and standard deviation (SD). The variable normality distributions were measured by the Shapiro–Wilk test. The significance of the variability of health behaviours in the sten scale was assessed with the chi-square test (χ^2^). The variance of the variables related to health behaviours and quality of life was assessed using the ANOVA (F) analysis of variance with the Brown–Forsythe test for homogeneity of variance. The Pearson correlation (r) was employed to assess the significance of the direction and strength of the correlation between the variables under analysis. The strength of the correlation between the analysed variables was interpreted based on Guilford’s classification: |r| = 0—no correlation; 0.0 < |r| ≤ 0.1—slight correlation; 0.1 < |r| ≤ 0.3—weak correlation; 0.3 < |r| ≤ 0.5—average correlation; 0.5 < |r| ≤ 0.7—high correlation; 0.7 < |r| ≤ 0.9—very high correlation; 0.9 < |r| < 1.0—nearly complete correlation; |r| = 1—complete correlation [41]. Predictors of quality of life were sought using multiple regression analysis by the progressive step method [41]. The level of significance of *p* < 0.05 was adopted.

## 3. Results

### 3.1. Participants

In total, 796 students at the University of Warmia and Mazury in Olsztyn participated in the study, including 684 women (85.9%) and 112 men (14.1%). The mean age of the participants was 20.7 years (SD = 1.7). There were 337 (42.3%) first-year students, 197 (24.8%) second-year students and 262 (32.9%) third-year students. Nearly half of the respondents (*n* = 380; 47.7%) lived with their family or with someone close, while the others lived in dormitories (25.9%, *n* = 206) or on their own (26.4%, *n* = 210). They spent more than 5.8 h (SD = 2.7 h) a day working on a computer on average. Nearly all of the students declared being satisfied with their health status. More than 70% (*n* = 571) of the respondents reported that they ate three to four meals a day, but usually not at the same time every day. One-third of the respondents did not reduce their physical exercise because of the COVID-19 pandemic, whereas 19.5% of them reduced it to a small extent, 23.2% reduced it to a medium extent and 24% reduced it to a considerable extent. When choosing a form of physical exercise, they usually decided to go walking or jogging (*n* = 466; 58.5%) or cycling (*n* = 159; 20%). Over 90% of the respondents reported that they reduced their social contacts because of the COVID-19 pandemic to a medium or considerable extent. Detailed data are provided in Table 1.

### 3.2. Variance of Health Behaviour Scores (IZZ) and of the Quality of Life (WHOQoL-Bref) among Students in the Subgroups under Study

A statistical analysis with the Shapiro–Wilk test showed that the variable distribution did not differ significantly in most cases from the normal distribution. The ANOVA (F) analysis of variance with the Brown–Forsythe homogeneity test revealed differences in the general intensity of pro-health behaviours between students in subgroups 1 and 2 (F = 44.92; <0.001). Students in subgroup 1 had a significantly higher general index of health behaviours—77 points (SD = 15) on a scale from 24 to 120—than the students in subgroup 2 (M = 70; SD = 13). The health behaviour intensity index was converted to standardised units during further analyses. These units were interpreted according to the properties of the sten scale. It was observed that during the COVID-19 pandemic, more than half of the students surveyed scored between 1 and 4 sten, accepted as low scores, but the distribution of the structure of health behaviours on the sten scale was significantly different among the subgroups surveyed (χ^2^ = 35.37; <0.001).

**Figure 1:**
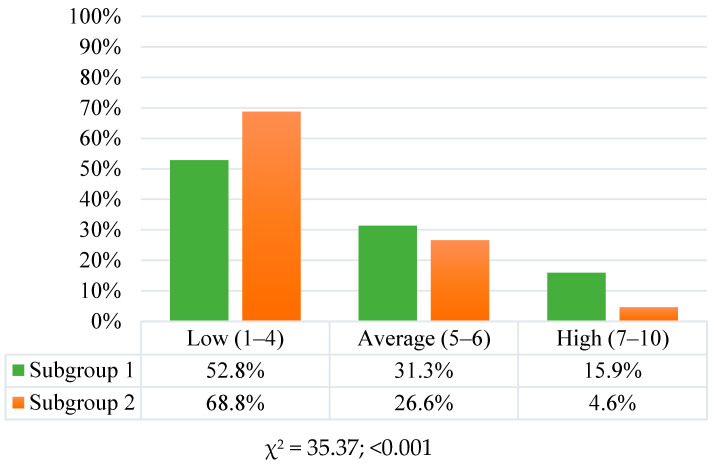
Distribution of health behaviour results on the sten scale in subgroup 1 (School of Public Health) and subgroup 2 (veterinary medicine).

A significantly higher percentage of students in subgroup 2 (68.8%) than in subgroup 1 (52.8%) had scores within the range of 1–4 sten, indicative of a low intensity of pro-health behaviours. Students in subgroup 1 demonstrated a high general intensity of health behaviours significantly more often (15.9%) than those in subgroup 2 (4.6%). The structure of health behaviour intensity for the two groups on the sten scale is shown in Figure 1.

Further statistical analyses estimated the intensity of health behaviours in another four categories. The subgroups differed statistically in terms of healthy eating habits, prophylactic behaviours, positive mental attitudes and health practices (Table 2). Students in subgroup 1 had significantly higher mean scores (M = 3.3; SD = 0.7) in the category of prophylactic behaviour associated with following health recommendations and obtaining information on one’s health and disease than students in subgroup 2 (M = 3.1; SD = 0.7). Regarding the health behaviour category referred to as a positive mental attitude, characterised by avoiding too strong emotions, stress and tensions, significantly lower mean scores (M = 3.2; SD = 0.7) were noted for students in subgroup 1 compared with those in subgroup 2 (M = 2.8; SD = 0.7) (Table 2).

Further statistical analyses showed that there were no significant differences in the way the overall quality of life and quality of health was perceived by students in the subgroups under study (Table 2). However, significant differences were observed in the perception of the quality of life in the domains: somatic, mental, social and environmental. Students in subgroup 1 had significantly higher scores compared with those in subgroup 2 in all of the analysed quality of life domains (Table 2).

### 3.3. Correlation between Preferred Health Behaviours and the Quality of the Students’ Lives

**Figure 2:**
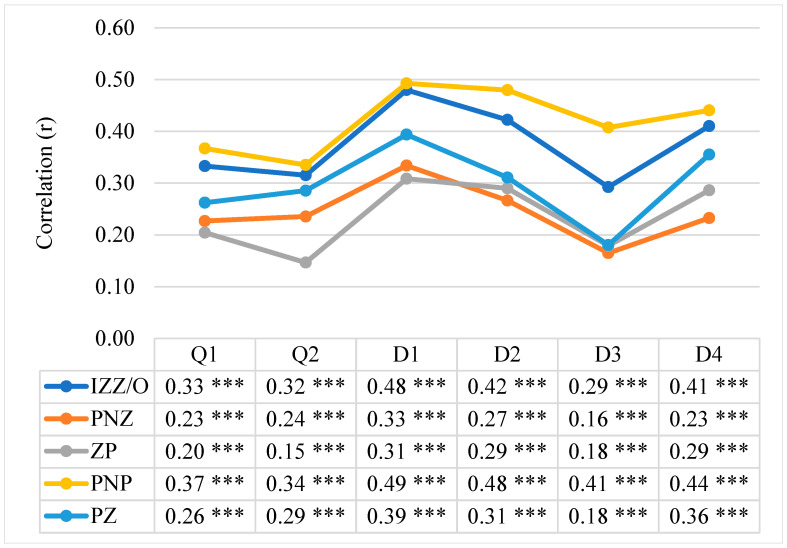
The nature and strength of correlations between the preferred health behaviours (IZZ) and the quality of life (WHOQoL-Bref) in all the students under study—Pearson correlation coefficient (r). Statistically significant: *** *p* < 0.001. Explanations: Q1—satisfaction with overall quality of life; Q2—satisfaction with overall quality of health; D1—somatic domain; D2—mental domain; D3—social domain (social relations); D4—environmental domain; IZZ/O—overall health behaviour level; PNZ—eating habits; ZP—prophylactic behaviours; PNP—positive mental attitude; PZ—health practices.

The next step involved the use of the Pearson linear correlation coefficient to determine the relationship between the health behaviour indices and the quality of the students’ lives, determining the strength and direction of the correlation. The correlation coefficients shown in Figure 2 are indicative of significant statistical links between health behaviours in general and in all the categories under analysis (i.e., proper eating habits, prophylactic behaviour, positive mental attitude, health practices) and the overall quality of life and quality of health, as well as the four domains of life quality (i.e., somatic, mental, social and environmental). These correlations are positive, which means that the higher the intensity of health behaviours, the higher the quality of students’ lives, and vice versa.

Considerable positive correlations (*p* < 0.001) on an average level are present between the overall health behaviour index and satisfaction with the overall quality of life (r = 0.33; *p* < 0.001) and of health (r = 0.32; *p* < 0.001), with the somatic (r = 0.48; *p* < 0.001), mental (r = 0.42; *p* < 0.001), environmental (r = 0.41; *p* < 0.001) domains and, at a weak level, with the social domain (r = 0.29; *p* < 0.001). It is noteworthy that the strongest positive correlations were observed between the “positive mental attitude” and the quality of life in the somatic (r = 0.49; *p* < 0.001), mental (r = 0.48; *p* < 0.001), social (r = 0.41; *p* < 0.001) and environmental domains (r = 0.44; *p* < 0.001). The data are shown in Figure 2.

### 3.4. Predictors for the Quality of Students’ Lives

In the statistical analyses that followed, an attempt was made to search for predictors of students’ quality of life. In building a multiple regression model using the progressive step method with an explained variable, quality of life as defined by the WHOQoL-Bref, and a pool of explanatory variables consisting of health behaviours, defined as eating habits, prophylactic behaviours, positive mental attitudes and health practices. The results of this study indicate that three variables, namely positive mental attitude, eating habits and health practices, were statistically significant predictors of quality of life for students in subgroup 1 (Table 3). However, the largest contribution was made by the health behaviour category defined as positive mental attitude, taking the following values (indicating explanation of variation in the results) in the individual WHOQoL-Bref domains: 19% in the somatic domain (ßeta = 0.24; R^2^ = 0.21), 20% in the mental domain (ßeta = 0.36; R^2^ = 0.20), 16% in the social domain (ßeta = 0.52; R^2^ = 0.17) and 17% in the environmental domain (ßeta = 0.19; R^2^ = 0.19). All regression coefficients were positive, indicating a positive relationship. In contrast, the variables such as eating habits and health practices, although they entered the regression model, did not play a significant role.

Subsequent regression analyses identified four variables, i.e., positive mental attitude, eating habits, health practices and prophylactic behaviours, which proved to be predictors of students’ quality of life in subgroup 2 (Table 4). The highest predictive power was contributed by the category of health behaviours defined as positive mental attitude, taking the following values (indicating explanation of variation in the results) in the individual WHOQoL-Bref domains: 23% in the somatic domain (ßeta = 0.24; R^2^ = 0.26), 25% in the mental domain (ßeta = 0.47; R^2^ = 0.25), 16% in the social domain (ßeta = 0.46; R^2^ = 0.17) and 21% in the environmental domain (ßeta = 0.38; R^2^ = 0.23). The effects of the analysis indicate that the other three variables that entered the regression model, defined as eating habits, health practices and prophylactic behaviours, explain only 1–2% of the variation in the results each, and this means that their power in predicting the quality of life of students in subgroup 2 is negligible. Detailed data are provided in Table 3 and Table 4.

## 4. Discussion

The COVID-19 pandemic presented new challenges for people as individuals and for the whole of society. The limitations aimed at containing the pandemic sometimes required considerable changes in people’s lifestyles and probably affected health behaviours. The pandemic had a negative impact on the quality of life of the whole population, especially with respect to physical, social, mental and spiritual health [42,43,44].

This study assessed the health behaviours of students in the context of their life quality during the COVID-19 pandemic. More than half of the students had low scores for health behaviours. However, significant differences were identified within the subgroups under study. The findings of a study by Zajacov et al. also indicate negative changes in health behaviours among adult Canadians during the COVID-19 pandemic [45]. The findings of studies conducted by other authors suggest that health behaviours deteriorated during a period of isolation caused by COVID-19, and this included a reduction in physical activity and the daily consumption of fruit and vegetables and an increase in the number of hours spent using a computer or a smartphone [13,46,47,48]. The period of isolation was linked with high mental pressure, which may have resulted in an increase in the amount and frequency of meals [49]. Similarly, Flaudias et al. observed that social isolation and related stress increased the risk of problematic eating behaviours among young people, e.g., compulsive eating [50]. It was confirmed in the ECLB-COVID-19 study that the products consumed and the eating patterns were more unhealthy during the period of isolation because of the pandemic than before it, whereas alcohol consumption decreased considerably during the pandemic [51]. The reduction in alcohol consumption may have resulted from a decrease in income and the closing of gastronomic facilities [52]. However, study findings are not consistent, since another study showed an increase in alcohol consumption, mainly by men, which may be a result of increased stress, job losses and lifestyle changes [53]. A study conducted by Czenczek-Lewandowska et al. showed that the COVID-19 pandemic brought about adverse changes in health behaviours and increased the sense of general anxiety in young adults. Study participants changed their eating preferences; they ate more sweets and cereal products, consumed more alcohol and fats and reduced their physical activity significantly. The generalised anxiety during the obligatory isolation had a negative impact on physical activity and the quality of sleep [54]. According to Acharya et al., the findings related to the impact of the pandemic on the health behaviours associated with sleep and eating are a cause for concern as they correlate with depression and anxiety symptoms [55]. It was also shown that physical activity alleviates symptoms of anxiety and depression, and it also improves one’s self-esteem and cognitive functions [56]. A meta-analysis conducted by Huang et al. confirmed that a sedentary lifestyle increases the risk of depression [57]. The COVID-19 pandemic favoured a sedentary lifestyle, as opportunities associated with spending actively were restricted. In this regard, the pandemic brought about highly disadvantageous conditions which may have long-term consequences [58].

This study also indicates a positive correlation between the respondents’ health behaviours and their quality of life. Moreover, it is demonstrated that a positive mental attitude was the most important predictor of quality of life among all health behaviours.

A study conducted by Orji et al. also showed health behaviours to be significantly correlated with the quality of life among adults in the USA [59]. The findings of a study conducted by Nari et al. suggest a link between a low score regarding a healthy lifestyle and a low quality of life, both in general and in the sphere of health [60]. A study conducted by Lee et al. confirmed lifestyle-related health behaviours to be linked with the predictive factors of the quality of life among teenagers [61]. Other studies additionally demonstrated a link between mental health and the quality of life among teenagers [62]. A study conducted by Szczepańska et al. showed that the COVID-19 pandemic-related restrictions brought about a considerable deterioration of mood, mental well-being and quality of life among young adults [63]. Researchers in other countries made similar observations [26,27,63,64,65,66,67,68,69,70]. According to Szczepańska et al., limited access to public spaces was strongly correlated with deterioration of mental well-being. According to respondents, a decrease in the frequency of visiting public spaces and a consequent decrease in physical and social activity had a highly negative impact on their mental well-being and mood. The students taking part in the survey conducted by Szczepańska et al. were almost unanimous in saying that their life quality had deteriorated as a result of social distancing [63]. A study conducted by Chusak et al. showed that more intensive use of a computer, tablet and smartphone in online learning was linked to shorter sleep and lower quality of life with respect to mental health [71]. These findings are consistent with other studies concerning online learning and mental health under COVID-19-related restrictions [72,73].

### Study Limitations and Implications for Professional Practice

This study has some limitations: the questionnaires did not contain questions about health behaviours and quality of life before COVID-19-related restrictions were imposed. Since there are no comparative data, this study could not assess the dynamics of changes in health behaviours and quality of life caused by the COVID-19 pandemic restrictions, and it could only assess these variables with respect to the pandemic.

The study findings provide new proof confirming links between health behaviours and quality of life among students during the COVID-19 pandemic. Identifying the quality of life predictors will allow for the development of educational programmes and social campaigns aimed at promoting health behaviours that have a beneficial impact on quality of life.

## 5. Conclusions

1. Over half of the students under study had low scores for health behaviours during the COVID-19 pandemic, but the distribution of the health behaviour structure in the sten scale was significantly different between the subgroups.
2. No significant differences were demonstrated regarding the perception of the overall quality of life and health in the subgroups under study, whereas students of medical majors had higher scores than veterinary students in the analysed quality of life domains.
3. Positive correlations between health behaviours and the quality of life among the study participants were determined. Pro-health behaviours had a beneficial impact on the respondents’ quality of life during the COVID-19 pandemic.
4. The category of health behaviours described as a positive mental attitude was an important predictor for the participants’ quality of life.

## Figures and Tables

**Table 1:** Sociodemographic variables.

Variables		Total		Subgroup 1		Subgroup 2		*p*-Value
		*n* = 796	%	*n* = 428	%	*n* = 368	%	
Sex	Female	684	85.9	381	89.	303	82.3	0.006
	Male	112	14.1	47	11.	65	17.7	
Year of studies	First	337	42.3	166	38.8	171	46.5	0.08
	Second	197	24.8	115	26.9	82	22.3	
	Third	262	32.9	147	34.3	115	31.3	
Age (years)	≤20	386	48.5	201	47.	185	50.3	0.009
	21	187	23.5	89	20.8	98	26.6	
	≥22	223	28.	138	32.2	85	23.1	
Stay/residence during COVID-19 pandemic	With a family/someone close	380	47.7	264	61.7	116	31.5	<0.001
	On their own	416	52.3	164	38.3	252	68.5	
Number of hours working on a computer	≤3 h	169	21.2	106	24.8	63	17.1	0.01
	4 h–7 h	407	51.1	202	47.2	205	55.7	
	≥8 h	220	27.6	120	28.	100	27.2	
Number of meals consumed	1–2	153	19.2	65	15.2	88	23.9	<0.001
	3	335	42.1	155	36.2	180	48.9	
	4	236	29.6	156	36.5	80	21.7	
	5 and more	72	9.1	52	12.1	20	5.4	
Degree of reduction in physical activity during COVID-19 pandemic	No reduction	265	33.3	144	33.6	121	32.9	0.85
	Slight	155	19.5	79	18.4	76	20.7	
	Medium	185	23.2	99	23.1	86	23.4	
	Significant	191	24.	106	24.8	85	23.1	

Explanation: *N*—total size of study group; *n*—size of distinguished subgroup; subgroup 1—School of Public Health students); subgroup 2—veterinary students).

**Table 2:** Variance of health behaviour scores and of the quality of life (WHOQoL-Bref) in the subgroups under study—a comparative analysis (*N* = 796).

Variables		Subgroup 1 *n* = 428 (53.8%)	Subgroup 2 *n* = 368 (46.2%)	*ANOVA* (*F*)	*p*-Value
		M ± SD	M ± SD		
Overall Health Behaviour		77 ± 15	70 ± 13	44.92	<0.001
Health behaviour components	Eating habits	3.2 ± 0.8	2.9 ± 0.8	26.29	<0.001
	Prophylactic behaviours	3.3 ± 0.7	3.1 ± 0.7	19.48	<0.001
	Positive mental attitude	3.2 ± 0.7	2.8 ± 0.7	51.65	<0.001
	Health practices	3 ± 0.8	2.8 ± 0.6	18.88	<0.001
Satisfaction with overall quality of life		3.7 ± 0.7	3.6 ± 0.9	3.06	0.08
Satisfaction with overall quality of health		3.6 ± 0.9	3.5 ± 1	2.94	0.09
WHOQoL-Bref domains	Somatic	11 ± 2	10 ± 2	59.96	<0.001
	Mental	13 ± 2	13 ± 2	10.60	0.001
	Social	15 ± 3	15 ± 3	8.45	0.004
	Environmental	14 ± 2	14 ± 2	5.47	0.02

Statistically significant: *p* < 0.05; *p* < 0.01; *p* < 0.001. M—arithmetic mean; SD—standard deviation.

**Table 3:** Predictors for the quality of students’ lives—subgroup 1.

Variables		R^2^	ßeta	ß	ß Error	t	*p*-Value
Satisfaction with overall quality of life	Positive mental attitude	0.1	0.26	0.26	0.05	5.04	<0.001
	R = 0.32; R^2^ = 0.11 corrected R^2^ = 0.10						
Satisfaction with overall quality of health	Positive mental attitude	0.13	0.29	0.37	0.08	4.63	<0.001
	Health practices	0.01	0.17	0.22	0.07	2.91	0.004
	R = 0.39; R^2^ = 0.15 corrected R^2^ = 0.14						
Somatic	Positive mental attitude	0.19	0.24	0.63	0.19	3.27	0.001
	Eating habits	0.02	0.24	0.03	0.01	3.23	0.001
	R = 0.46; R^2^ = 0.21; corrected R^2^ = 0.21						
Mental	Positive mental attitude	0.20	0.36	1.03	0.22	4.79	<0.001
	R = 0.44 R^2^ = 0.20, corrected R^2^ = 0.20						
Social	Positive mental attitude	0.16	0.52	2.19	0.32	6.82	<0.001
	Eating habits	0.01	−0.15	−0.03	0.02	−2.01	0.04
	R = 0.40; R^2^ = 0.17; corrected R^2^ = 0.17						
Environmental	Positive mental attitude	0.17	0.19	0.56	0.27	2.05	0.04
	Eating habits	0.01	0.35	0.05	0.02	2.50	0.01
	R = 0.43; R^2^ = 0.19; corrected R^2^ = 0.18						

R—correlation coefficient; R^2^—multiple determination coefficient; ßeta—standardised regression coefficient; ß—non-standardised regression coefficient; Error ß—non-standardised regression coefficient error; t—*t*-test value.

**Table 4:** Predictors for the quality of students’ lives—subgroup 2.

Variables		R^2^	ßeta	ß	ß Error	t	*p*-Value
Satisfaction with overall quality of life	Positive mental attitude	0.18	0.33	0.40	0.09	4.39	<0.001
	R = 0.42; R^2^ = 0.8; corrected R^2^ = 0.18						
Satisfaction with overall quality of health	Positive mental attitude	0.09	0.23	0.32	0.09	3.60	0.001
	Eating habits	0.02	0.22	0.27	0.07	3.83	0.001
	Prophylactic behaviours	0.01	−0.15	−0.22	0.09	−2.59	0.01
	R = 0.36; R^2^ = 0.13 corrected R^2^ = 0.12						
Somatic	Positive mental attitude	0.23	0.24	0.64	0.21	3.02	0.001
	Eating habits	0.02	0.40	0.06	0.02	3.71	0.001
	Prophylactic behaviours	0.01	−0.17	−0.46	0.20	−2.23	0.03
	R = 0.51; R^2^ = 0.26; corrected R^2^ = 0.25						
Mental	Positive mental attitude	0.25	0.47	1.49	0.15	9.67	<0.001
	R = 0.50 R^2^ = 0.25, corrected R^2^ = 0.25						
Social	Positive mental attitude	0.16	0.46	2.08	0.26	7.98	<0.001
	Health practices	0.01	−0.12	−0.60	0.29	−2.05	0.04
	R = 0.41; R^2^ = 0.17; corrected R^2^ = 0.17						
Environmental	Positive mental attitude	0.21	0.38	1.15	0.17	6.83	<0.001
	Health practices	0.01	0.14	0.48	0.19	2.58	0.01
	R = 0.47; R^2^ = 0.23; corrected R^2^ = 0.22						

R—correlation coefficient; R^2^—multiple determination coefficient; ßeta—standardised regression coefficient; ß—non-standardised regression coefficient; Error ß—non-standardised regression coefficient error; t—*t*-test value.

## Data Availability

Data are contained within the article.
